# Antimicrobial Drug Consumption on Swiss Pig Farms: A Comparison of Swiss and European Defined Daily and Course Doses in the Field

**DOI:** 10.3389/fvets.2019.00240

**Published:** 2019-07-18

**Authors:** Thomas Echtermann, Cedric Muentener, Xaver Sidler, Dolf Kümmerlen

**Affiliations:** ^1^Division of Swine Medicine, Department for Farm Animals, Vetsuisse Faculty, University of Zurich, Zurich, Switzerland; ^2^Institute of Veterinary Pharmacology and Toxicology, Vetsuisse Faculty, University of Zurich, Zurich, Switzerland

**Keywords:** antimicrobial drug usage, antimicrobial classes, defined daily dose, defined course dose, European medicines agency, monitoring systems, pigs, Switzerland

## Abstract

Defined Daily Doses (DDD) and Defined Course Doses (DCD) have been established in both human and veterinary medicine in order to standardize the measurement of treatments in a population. In 2016 the European Medicines Agency published average defined daily dose (DDDvet) and defined course dose (DCDvet) values for antimicrobial agents used in livestock production. Similarly, national defined doses (DDDch and DCDch) for the pig sector in Switzerland have recently been determined. The aim of this study was to compare the outcome of calculating antimicrobial consumption based on either DDDvet/DCDvet or DDDch/DCDch. Data from 227 Swiss pig farms describing antimicrobial use in 2015 was collected. The numbers of treatment days and treatments were calculated using DDDvet/DCDvet and DDDch/DCDch respectively, for each farm in total and for different antimicrobial classes. Associations between calculated numbers of DDDvet/DCDvet and DDDch/DCDch on farm level were investigated. In addition, differences concerning antimicrobial use were investigated between different production types of farms (piglet-producer, finishing farm or farrow-to-finishing farm). Using DDDch/DCDch values we calculated 1,805,494 treatment days and 433,678 treatments compared to 1,456,771 treatment days (19% ratio) and 303,913 treatments (30% ratio) based on DDDvet/DCDvet. Penicillins (21.4/26.6%), polypeptides (18.6/27.6%) and fluoroquinolones (9.5/8.8%) were the most frequently used classes of antimicrobials based on calculation using both DDDch and DDDvet. Similar findings were observed for complete treatments (DCDch/vet) (penicillins: 52.8/39.6%; polypeptides: 7.8/14.2%; fluoroquinolones: 13.2/12.9%). The number of treatment days or treatments per farm was higher for piglet-producers and farrow-to-finishing farms compared to finisher farms regardless of whether Swiss or European DDD or DCD values were used for the calculation (each *P* < 0.001). Similar results for antimicrobial use (AMU) obtained at farm level were observed when calculated either by Swiss or European definitions. Nevertheless, marked differences could be observed in the assessment of the use of specific antimicrobial classes in the field based on DDDvet/DCDvet compared to DDDch/DCDch.

## Introduction

Antimicrobial use (AMU) is associated with the selection of resistant pathogens (1, 2) and the spread of resistance both within and between human and veterinary medicine (3–5). Responsible use of antimicrobials is therefore essential (6).

The importance of antimicrobial resistance for public health is internationally acknowledged (7, 8) and AMU in food-producing animals is monitored by various authorities (9, 10) in order to determine trends in resistance development.

In addition to monitoring systems measuring the amount of active ingredients, systems based on application equivalents have been established in several countries to monitor AMU in veterinary medicine (11–13). These application equivalents, originally developed for humans (14), standardize the measurement of AMU (15), by taking into account the dosages of the various antimicrobial compounds, and defining a dosage required daily or for a whole treatment. In line with the formal definition of the World Health Organization (WHO), Defined Daily Doses (DDD) and Defined Course Doses (DCD) are the assumed average maintenance doses per day or total treatment duration (16), which allow the estimation of number of treatment days respectively, number of treatments in a population (17).

In 2016, following these principles, the European Medicines Agency (EMA) published average defined daily dose (DDDvet) and defined course dose (DCDvet) values for antimicrobial agents used in livestock production as a tool to facilitate standardized collection and presentation of AMU among European member states (18). These values were defined by calculating the mean of given registrations for livestock production from nine different European countries. In analogy with the principles of the EMA (19), national defined doses (DDDch and DCDch) for each individual registration in the pig sector in Switzerland were recently determined and some theoretically discrepancies between Swiss and European values have been described in a prior study (20).

The aim of this study was to investigate the outcome of calculated antimicrobial consumption in the field based on either individual, Swiss values (DDDch/DCDch) or average, European values (DDDvet/DCDvet). The impact of using either DDD/DCDch or DDD/DCDvet values were tested for different age groups, administration routes and antimicrobial classes. Moreover, the impact of using either DDD/DCDch or DDD/DCDvet for evaluation of antimicrobial use on the study farms was considered, as well as differences in antimicrobial usage by farm type.

The questions behind all these investigations were: Will an AMU monitoring system based on either Swiss or European definitions lead to comparable results in the field or not? And for which age groups, administration routes and antimicrobial classes can the most frequent AMU be observed in the pig sector of Switzerland?

## Materials and Methods

### Data Collection

In cooperation with the Swiss Swine Health Service (SSHS), data from 227 Swiss pig farms concerning antimicrobial use in 2015 was collected, thus representing 3.3% of all pig farms in Switzerland in 2015 (21). All 227 farms joined a nationwide voluntary program for pig producers in Switzerland in order to evaluate and improve transparency of AMU on their farms. Only farms with a complete documentation of antimicrobial ingredients purchased in the year 2015 were included in the study. The study farms were required to provide documentation of all veterinary prescriptions of antimicrobials for this year, including exact information about the name and the amount of the used products. Farmers were required to allocate the prescribed antimicrobials to four different groups (sow, finisher pig, weaner, and piglet). The documentation had to be reported once every 3 months during the year. In addition to AMU records, numbers of pigs kept (sows) or produced annually (all other age groups) and the type of farm were also documented. Overall 96 piglet-producing farms, 42 farrow-to-finish pig farms and 89 finishing farms housing a total of 328,909 piglets, 292,298 weaners, 179,144 finishing pigs and 11,710 sows were included in the study. The number of sows were representing 9.5% of all sows kept in Switzerland, which were notified in 2015 (21). The mean farm size was 85 sows with 2,383 produced piglets and 2,108 produced weaners in the year 2015, including the data of all piglet-producing and farrow-to-finish farms. The mean of the produced finishing pigs was 1,303, combined the data of the farrow-finish farms and the finishing farm, respectively. A piglet-producing farm housing at least 30% piglets from birth until time of slaughter was considered as farrow-to-finish farm.

### AMU Quantification

In order to quantify AMU, the amount of prescribed antimicrobial ingredient during the year 2015 of all participating farms was divided by the defined doses (DDD/DCDvet or DDD/DCDch) of the corresponding antimicrobial classes multiplied by the standard weights of the different age groups as defined by the European Surveillance of Veterinary Antimicrobial Consumption (ESVAC) (piglets: 4 kg; weaners: 12 kg; finisher pig: 50 kg and sow: 220 kg) (22).

$$Number\;of\;Defined\;Doses=\frac{total\;amount\;of\;prescribed\;antimicrobial\;ingredient\;(mg)}{DDDvet\;or\;DDDch\;or\;DCDvet\;or\;DCDch\;\left(\frac{mg}{kg}\right)\times standard\;weight\;of\;age\;group\;(kg)}\;$$

The recently published, national defined daily and course doses for the pig sector in Switzerland were drawn up in accordance with the principles of the EMA (19). In order to establish DDDch and DCDch, the required information on dosage and treatment duration was generally taken from the product approvals which are summarized in the Swiss Veterinary Medicines Compendium (23). The detailed procedure of defining the national doses and all values for DDDch and DCDch is accessible as Supplementary Data. A product and the farm using it were excluded from the study when corresponding DDD/DCDvet values had not been published by the EMA either for the specific antimicrobial ingredient or a comparable antimicrobial ingredient, a given combination of substances or a specific administration route.

The number of defined doses and the amount of prescribed antimicrobial ingredient were calculated in total, for the different age groups, different administration routes (injection, oral, and premix) and for all antimicrobial classes. The term premix included all antimicrobial ingredients to be administered via the feed and/or water. By dividing the results using DDD/DCDvet by those based on DDD/DCDch, differences of Swiss or European definitions were investigated for the calculated AMU. The results of this calculations were termed ratio. A positive ratio with results > 0 indicated a higher number of estimated treatment days or treatments could be observed when using the European definitions DDD vet or DCDvet. In addition, the overall observed mean treatment durations given by the Swiss or European defined values were compared in the same way.

For the evaluation of the AMU at the farm level and in order to compare the consumption on different farms, the number of kept (sows) or produced pigs (other age groups) in the year 2015 were taken into account. The amount of prescribed antimicrobial ingredients was divided by the different defined doses, the standard weights and the number of pigs for each age group. If necessary, the results of the different age groups were summarized together and the number of Defined Doses per farm was calculated.

$$Number\;of\;Defined\;Doses\;per\;farm=\frac{total\;amount\;of\;prescribed\;antimicrobial\;ingredient\;per\;farm\;and\;age\;group\;(mg)}{DDDvet\;or\;DDDch\;or\;DCDvet\;or\;DCDch\;\left(\frac{mg}{kg}\right)\times standard\;weight\;of\;age\;group\;(kg)\times number\;of\;pigs\;per\;farm\;and\;age\;group}\;$$

### Data Processing and Statistical Analysis

The preparation of all operating farm data and the calculation of the number of defined doses was carried out using Microsoft Excel 2011 (Microsoft, Redmond, WA, USA). The statistical analysis and preparation of graphs to visualize the results was performed with R (https://cran.r-project.org). Differences between the tested groups having a *P* ≤ 0.05 were considered statistically significant. The data for calculated AMU on farm level was tested for normality by the Shapiro-Wilk test. The association of Swiss and European dosages for a possible AMU monitoring system on the farms was evaluated using scatterplots and correlation analysis performed by Spearman's rho test. The differences between the various farm structures were investigated using the Kruskal-Wallis test for independent samples and *post hoc* pairwise analysis (Bonferroni correction).

## Results

### AMU Quantification per Age Group and Administration Route

In this study, the AMU was calculated at 1,805,494 DDDch and 433,678 DCDch when based on Swiss values, compared to 1,456,771 DDDvet (−19.3% ratio) and 303,913 DCDvet (−29.9% ratio) based on European defined doses (Table 1). The mean treatment duration was 3.7 days based on Swiss values and 4.0 days based on European values. The largest fraction of DDD was calculated for weaners, regardless of Swiss DDDch (64.4%) or European DDDvet (60.3%), whereas for DCDs based on Swiss definitions, piglets represented the major part of the treatments (53.1%). Based on European definitions most calculated course doses were observed for weaners (44.8%). Ratios of more than 20% between the calculated numbers of DDD/DCDch and DDD/DCDvet, respectively could be observed for the number of DDDch/vet of weaners and finisher pigs and for DCDch/vet of piglets, finisher pigs, and sows. The largest quantity of active ingredients was given to the groups of weaners (49.4%), followed by finisher pigs (32.7%), sows (14.2%), and piglets (3.6%). When investigating the different administration routes by the number of defined doses, premixes represented the largest proportion calculated by DDDch/vet (64.9/59.5%) in contrast to injectable products when calculating the number of DCDch/vet (71.3/60.2%). Relative differences of more than ±20% could be observed for oral and premix treatments when calculated in DDDch and DDDvet, respectively, and for oral and parenteral treatments when calculated in DCDch or DCDvet. The treatment duration was longer when the calculation was based on DDD/DCDvet compared to DDD/DCDch, except for treatments of weaners in general and for treatments with premixes.

**Table 1:** Total and relative antimicrobial use (AMU) on 227 Swiss pig farms in the year 2015.

	**Amount of active ingredient in kg (percent)**	**DDDch^**a**^ (percent)**	**DDDvet^**b**^ (percent)**	**Ratio**	**DCDch^**c**^ (percent)**	**DCDvet^**d**^ (percent)**	**Ratio**	**treatment duration ch**	**treatment duration vet**	**ratio**
Overall Result	421	1,805,494	1,456,771	−19.3%	433,678	303,913	**−29.9%**	3.7	4.0	8.1%
**AGE GROUP**										
Piglets	15 (3.6%)	473,922 (26.3%)	428,546 (29.4%)	−9.6%	230,237 (53.1%)	132,433 (43.6%)	**−42.5%**	2.9	3.5	**20.7%**
Weaners	208 (49.4%)	1,143,175 (63.3%)	878,525 (60.3%)	**−23.2%**	151,483 (34.9%)	136,136 (44.8%)	−10.1%	5.6	5.0	−10.7%
Finisher pigs	138 (32.8%)	159,719 (8.8%)	122,493 (8.4%)	**−23.3%**	40,894 (9.4%)	27,566 (9.1%)	**−32.6%**	3.7	4.0	8.1%
Sows	60 (14.2%)	28,678 (1.6%)	27,207 (1.9%)	−5.1%	11,064 (2.5%)	7,778 (2.6%)	**−29.7%**	2.9	3.6	**24.1%**
**ADMINISTRATION ROUTE**										
Oral	0,1 (0.0%)	13,856 (0.7%)	10,080 (0.7%)	**−27.2%**	4,435 (1.0%)	2,432 (0.8%)	**−45.2%**	3.3	4.2	**27.3%**
Injection	114 (27.1%)	620,458 (34.4%)	579,947 (39.8%)	−6.5%	309,277 (71.3%)	182,839 (60.2%)	**−40.9%**	2.7	3.4	**25.9%**
Premix	307 (72.9%)	1,171,180 (64.9%)	866,744 (59.5%)	**−26.0%**	119,966 (27.7%)	118,642 (39.0%)	−1.1%	9.9	7.6	**−23.2%**

AMU is measured as active ingredient and by Swiss and European defined dosage grouped by age category and administration route. The ratio between the number of treatment days and treatments, as well as the overall observed mean treatment durations based on Swiss doses or the doses of the European Medicine Agency (EMA) was calculated. Numbers in bold shown a ratio > ±20%.

a DDDch: Number of treatment days based on Swiss Defined Daily Doses.

b DDDvet: Number of treatment days based on Defined Daily Doses of the European Medicine Agency (EMA).

c DCDch: Number of treatments based on Swiss Defined Course Doses.

d DCDvet: Number of treatments based on Defined Course Doses of the European Medicine Agency (EMA).

### AMU Quantification per Antimicrobial Classes

**Figure 1:**
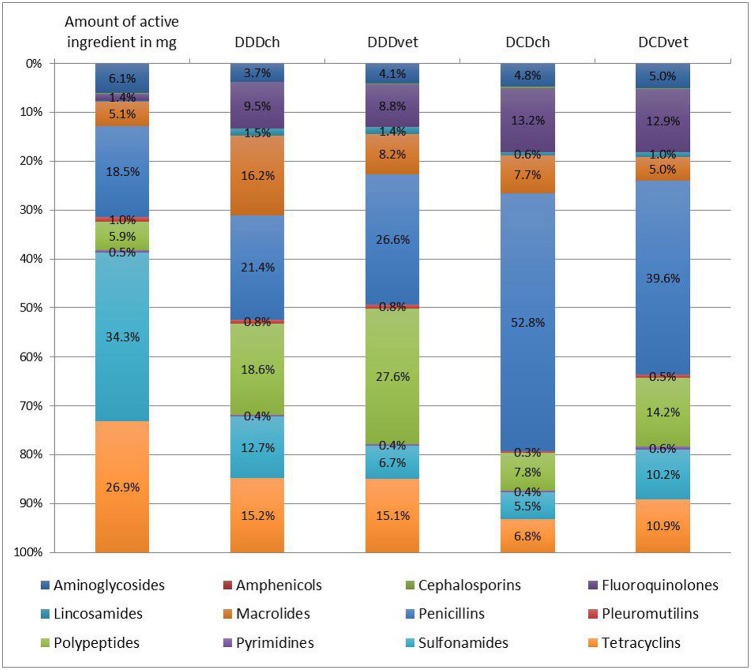
Relative distribution of antimicrobial use (AMU) between different antimicrobial classes measured either as the amount of active ingredient or as the number of defined daily doses (DDD) or defined course doses (DCD), respectively. DDD and DCD were calculated with Swiss values (DDDch and DCDch) or European values (DDDvet or DCDvet) published by the European Medicine Agency (EMA). (Amphenicols and cephalosporins as well as lincosamides are not inscribed due to the low values).

The amount of active ingredient and the calculated numbers of defined doses for different antimicrobial classes were summarized in Table 2 and the relative distribution was visualized in Figure 1. Considering the amount of active ingredient used, the classes of sulfonamides (144,086,000 mg/34.3%), tetracyclines (113,122,600 mg/26.9%), and penicillins (77,788,850 mg/18.5%) represented the largest proportion of the total usage, whereas when using defined daily doses, penicillins (DDDch: 385,507/21.4%; DDDvet: 388,221/26.6%) and polypeptides (DDDch: 335,498/18.6%; DDDvet: 402,708/27.6%) were the most frequent. Macrolides were observed to represent 16.2% of the total usage (293,108 treatment days) calculated in DDDch. Penicillins (DCDch: 229,006/52.8%; DCDvet: 120,394/39.6%) and fluroquinolones (DCDch: 57,173/13.2%; DCDvet: 39,064/12.9%) were common for the number of total treatments, as well as polypeptides (DCDvet: 43,006/14.2%) for calculations based on the European values. The percentage of fluoroquinolones in total AMU was 1.4% when considering the amount in mg, compared to 8.8 and 13.2% when calculating DDDvet and DCDch, respectively.

**Table 2:** Total antimicrobial use (AMU) measured as active ingredient and by Swiss and European defined dosage grouped by different antimicrobial classes.

**Antimicrobial classes**	**Amount of active ingredient in kg**	**DDDch^**a**^**	**DDDvet^**b**^**	**DCDch^**c**^**	**DCDvet^**d**^**
Aminoglycosides	25.7	67,273	59,973	20,918	15,255
Amphenicols	0.03	44	69	22	22
Cephalosporins	0.3	2,200	2,299	733	636
Fluoroquinolones	6.0	171,518	127,880	57,173	39,064
Lincosamides	0.7	26,217	20,456	2,777	2,997
Macrolides	21.4	293,108	120,006	33,286	15,148
Penicillins	77.8	385,507	388,221	229,006	120,394
Pleuromutilins	4.0	14,388	11,289	1,188	1,623
Polypeptides	26.0	335,498	402,708	33,687	43,006
Pyrimidines	2.1	6,613	6,252	1,653	1,705
Sulfonamides	144.1	228,817	98,192	23,946	30,848
Tetracyclins	113.1	274,311	219,426	29,289	33,215

a DDDch: Number of treatment days based on Swiss Defined Daily Doses.

b DDDvet: Number of treatment days based on Defined Daily Doses of the European Medicine Agency (EMA).

c DCDch: Number of treatments based on Swiss Defined Course Doses.

d *DCDvet: Number of treatments based on Defined Course Doses of the European Medicine Agency (EMA)*.

A more detailed, combined consideration of age groups, administration routes, and antimicrobial class data shows that injection was the most frequent administration route for piglets independent of the method used for calculation, and that within this group penicillins and fluoroquinolones were the most frequently used antimicrobials (Table 3). The use of premixes was the most frequently used administration route for weaners independent of the indicator used and polypeptides were most frequently used when considering the number of defined daily doses. For the number of calculated doses based on DDD/DCDch, frequent use of macrolides was notable in the premixes given to weaners whereas sulfonamides and tetracyclines were more frequently used when the calculation was based on DDD/DCDvet. In terms of the finisher pig group, injection and premixes were observed with similar frequencies for administration routes, when either daily doses or course doses were the basis of the calculation. Oral administration of premixes was the most common administration route when calculating AMU based on defined doses. Contrastingly, when calculating in course doses, injections represented the largest proportion of treatments. Penicillins and aminoglycosides were frequently used injections for finisher pigs and tetracyclines were the most commonly used antimicrobial class given as premix. As was the case in weaners, macrolides represented a considerable proportion of treatments based on DDDch as well as DCDch. In sows, most antimicrobials were given by injections and within this group, most of the antimicrobials administered belonged to the antimicrobial classes of penicillins and fluoroquinolones. The most frequently administered antimicrobial class provided as a premix was the class of penicillins. An administration of oral antimicrobials without feed or water was only observed for fluoroquinolones and polypeptides in piglets and on only two farms with a small amount in weaners.

**Table 3:** Distribution of antimicrobial use (AMU) per different age categories, administration routes and antimicrobial classes measured as active ingredient and by Swiss and European defined dosage.

**Age group**	**Administration route**	**Antimicrobial classes**	**Amount of active ingredient in mg**		**DDDch^**a**^ (*n*)**	**(%)**	**DDDvet^**b**^ (*n*)**	**(%)**	**DCDch^**c**^ (*n*)**	**(%)**	**DDDvet^**d**^ (*n*)**	**(%)**
Piglets			15,117,075		473,922		428,546		230,237		132,433	
	Oral		118,250	0.8%	13,833	2.9%	10,065	2.3%	4,428	1.9%	2,428	1.8%
		Fluoroquinolones	83,050	70.2%	12,458	90.1%	8,305	82.5%	4,153	93.8%	2,076	85.5%
		Polypeptides	35,200	29.8%	1,375	9.9%	1,760	17.5%	275	6.2%	352	14.5%
	Injection		14,764,825	**97.7%**	450,340	**95.0%**	406,781	**94.9%**	224,835	**97.7%**	128,760	**97.2%**
		Aminoglycosides	2,662,850	18.0%	39,802	8.8%	36,445	9.0%	13,419	6.0%	9,661	7.5%
		Cephalosporins	7,875	0.1%	880	0.2%	916	0.2%	293	0.1%	259	0.2%
		Fluoroquinolones	1,281,725	8.7%	129,323	**28.7%**	95,830	**23.6%**	43,108	**19.2%**	29,632	**23.0%**
		Lincosamides	297,500	2.0%	14,875	3.3%	9,535	2.3%	2,125	0.9%	2,010	1.6%
		Macrolides	98,000	0.7%	2,450	0.5%	1,885	0.5%	817	0.4%	471	0.4%
		Penicillins	10,118,275	**68.5%**	257,065	**57.1%**	253,251	**62.3%**	163,096	**72.5%**	83,993	**65.2%**
		Pleuromutilins	20,000	0.1%	400	0.1%	417	0.1%	133	0.1%	227	0.2%
		Pyrimidines	22,000	0.1%	1,857	0.4%	1,833	0.5%	464	0.2%	500	0.4%
		Sulfonamides	110,000	0.7%	1,857	0.4%	1,782	0.4%	464	0.2%	480	0.4%
		Tetracyclins	146,600	1.0%	1,833	0.4%	4,887	1.2%	916	0.4%	1,527	1.2%
	Premix		234,000	1.5%	9,750	2.1%	11,700	2.7%	975	0.4%	1,245	0.9%
		Polypeptides	234,000	100.0%	9,750	100.0%	11,700	100.0%	975	100.0%	1,245	100.0%
Weaners			207,658,150		1,143,175		878,525		151,483		136,136	
	Oral		450	0.0%	23	0.0%	15	0.0%	8	0.0%	4	0.0%
		Fluoroquinolones	450	100.0%	23	100.0%	15	100.0%	8	100.0%	4	100.0%
	Injection		8,654,000	4.2%	82,799	7.2%	89,073	10.1%	42,440	28.0%	28,684	21.1%
		Aminoglycosides	755,300	8.7%	5,946	7.2%	4,621	5.2%	1,790	4.2%	1,252	4.4%
		Cephalosporins	12,500	0.1%	486	0.6%	508	0.6%	162	0.4%	141	0.5%
		Fluoroquinolones	567,400	6.6%	21,078	25.5%	16,860	18.9%	7,026	16.6%	5,237	18.3%
		Lincosamides	62,500	0.7%	1,042	1.3%	668	0.7%	149	0.4%	141	0.5%
		Macrolides	100,000	1.2%	833	1.0%	641	0.7%	278	0.7%	160	0.6%
		Penicillins	5,955,400	68.8%	46,766	56.5%	52,806	59.3%	30,355	71.5%	17,753	61.9%
		Pleuromutilins	10,000	0.1%	67	0.1%	69	0.1%	22	0.1%	38	0.1%
		Pyrimidines	36,400	0.4%	1,264	1.5%	1,011	1.1%	316	0.7%	276	1.0%
		Sulfonamides	182,000	2.1%	1,264	1.5%	1,083	1.2%	316	0.7%	310	1.1%
		Tetracyclins	972,500	11.2%	4,052	4.9%	10,806	12.1%	2,026	4.8%	3,377	11.8%
	Premix		199,003,700	**95.8%**	1,060,353	**92.8%**	789,437	**89.9%**	109,036	**72.0%**	107,448	**78.9%**
		Aminoglycosides	268,400	0.1%	10,167	1.0%	6,578	0.8%	484	0.4%	828	0.8%
		Lincosamides	268,400	0.1%	10,167	1.0%	10,167	1.3%	484	0.4%	828	0.8%
		Macrolides	15,594,000	7.8%	264,722	**25.0%**	108,292	13.7%	29,222	**26.8%**	13,260	12.3%
		Penicillins	5,613,500	2.8%	22,598	2.1%	27,517	3.5%	4,520	4.1%	2,613	2.4%
		Pleuromutilins	400,000	0.2%	4,444	0.4%	3,436	0.4%	317	0.3%	412	0.4%
		Polypeptides	22,934,400	11.5%	318,533	**30.0%**	382,240	**48.4%**	31,853	**29.2%**	40,664	**37.8%**
		Sulfonamides	91,210,000	45.8%	200,556	18.9%	82,618	10.5%	20,056	**18.4%**	26,120	**24.3%**
		Tetracyclins	62,715,000	31.5%	229,167	21.6%	168,589	21.4%	22,099	**20.3%**	22,723	**21.1%**
Finisher pigs			137,539,345		159,719		122,493		40,894		27,566	
	Injection		36,317,845	26.4%	59,832	37.5%	58,296	47.6%	31,176	**76.2%**	17,751	**64.4%**
		Aminoglycosides	9,080,625	25.0%	8,386	**14.0%**	9,221	**15.8%**	3,872	**12.4%**	2,627	**14.8%**
		Amphenicols	32,700	0.1%	44	0.1%	69	0.1%	22	0.1%	22	0.1%
		Cephalosporins	31,500	0.1%	315	0.5%	332	0.6%	105	0.3%	89	0.5%
		Fluoroquinolones	108,125	0.3%	915	1.5%	713	1.2%	305	1.0%	220	1.2%
		Lincosamides	30,000	0.1%	120	0.2%	77	0.1%	17	0.1%	16	0.1%
		Macrolides	653,600	1.8%	1,307	2.2%	1,006	1.7%	436	1.4%	251	1.4%
		Penicillins	24,298,495	**66.9%**	46,202	**77.2%**	41,541	**71.3%**	25,360	**81.3%**	12,839	**72.3%**
		Pleuromutilins	90,000	0.2%	144	0.2%	150	0.3%	48	0.2%	82	0.5%
		Pyrimidines	58,000	0.2%	377	0.6%	387	0.7%	94	0.3%	105	0.6%
		Sulfonamides	290,000	0.8%	377	0.6%	414	0.7%	94	0.3%	129	0.7%
		Tetracyclins	1,644,800	4.5%	1,645	2.7%	4,386	7.5%	822	2.6%	1,371	7.7%
	Premix		101,221,500	73.6%	99,887	**62.5%**	64,197	**52.4%**	9,718	23.8%	9,815	35.6%
		Macrolides	4,897,000	4.8%	23,767	**23.8%**	8,162	12.7%	2,525	**26.0%**	999	10.2%
		Penicillins	1,950,000	1.9%	1,917	1.9%	2,294	3.6%	383	3.9%	218	2.2%
		Pleuromutilins	350,0000	3.5%	9,333	9.3%	7,216	11.2%	667	6.9%	864	8.8%
		Polypeptides	1,752,000	1.7%	5,840	5.8%	7,008	10.9%	584	6.0%	746	7.6%
		Sulfonamides	42,145,000	41.6%	21,567	21.6%	9,162	14.3%	2,210	22.7%	2,897	29.5%
		Tetracyclins	46,977,500	46.4%	37,464	**37.5%**	30,355	**47.3%**	3,349	**34.5%**	4,091	**41.7%**
Sows			59,835,480		28,678		27,207		11,064		7,778	
	Injection		54,560,980	**91.2%**	27,489	**95.9%**	25,797	**94.8%**	10,826	**97.8%**	7,644	**98.3%**
		Aminoglycosides	12,929,300	23.7%	2,972	10.8%	3,108	12.0%	1,352	12.5%	885	11.6%
		Cephalosporins	236,525	0.4%	519	1.9%	544	2.1%	173	1.6%	148	1.9%
		Fluoroquinolones	3,930,475	7.2%	7,722	**28.1%**	6,157	**23.9%**	2,574	**23.8%**	1,895	**24.8%**
		Lincosamides	15,000	0.0%	14	0.0%	9	0.0%	2	0.0%	2	0.0%
		Macrolides	62,000	0.1%	28	0.1%	22	0.1%	9	0.1%	5	0.1%
		Penicillins	24,578,680	**45.0%**	9,769	**35.5%**	9,401	**36.4%**	5,054	**46.7%**	2,844	**37.2%**
		Pyrimidines	1,993,800	3.7%	3,116	11.3%	3,021	11.7%	779	7.2%	824	10.8%
		Sulfonamides	10,149,000	18.6%	3,197	11.6%	3,133	12.1%	806	7.4%	914	12.0%
		Tetracyclins	666,200	1.2%	151	0.6%	404	1.6%	76	0.7%	126	1.7%
	Premix		5,274,500	8.8%	1,189	4.1%	1,410	5.2%	238	2.2%	134	1.7%
		Penicillins	5,274,500	100.0%	1,189	100.0%	1,410	100.0%	238	100.0%	134	100.0%

Numbers in bold are mentioned in the results part of the study.

a DDDch: Number of treatment days based on Swiss Defined Daily Doses.

b DDDvet: Number of treatment days based on Defined Daily Doses of the European Medicine Agency (EMA).

c DCDch: Number of treatments based on Swiss Defined Course Doses.

d *DCDvet: Number of treatments based on Defined Course Doses of the European Medicine Agency (EMA)*.

### AMU Monitoring on Farm Level

Each dataset was tested for normality by Shapiro-Wilk test and for all datasets, independent of Swiss or European measuring method or type of farm, the null hypothesis was rejected (each *P* < 0.001).

**Figure 2:**
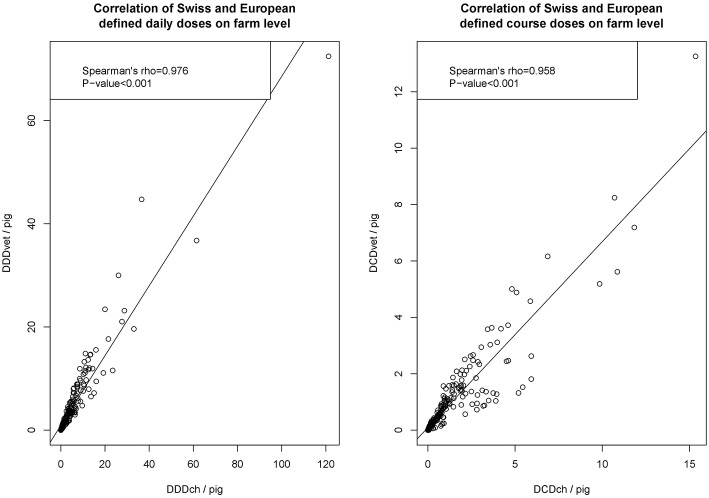
Scatterplots of defined daily doses (DDD) and defined course doses (DCD) at the farm level calculated either by Swiss values (DDDch/farm and DCDch/farm). Each dataset was tested for normality by Shapiro-Wilk test and for all datasets the null hypothesis was rejected (each *P* < 0.001). So non-normal distributed data was concluded and the correlation was investigated by Spearman's rho test.

The scatterplot of calculated defined daily doses (DDD) and defined course doses (DCD), analyzing the association between Swiss (ch) and European (vet) definitions, is given in Figure 2. As shown, both the calculated number of daily doses and the calculated number of course doses showed a positive correlation between results on the farm level by Spearman's rho test.

**Figure 3:**
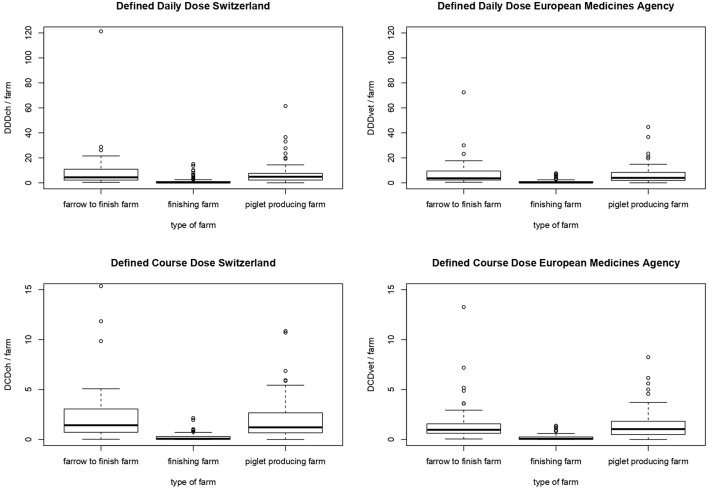
Comparison of antimicrobial use in different types of farms (farrow-to-finish farms, finishing farms and piglet-producing farms) measured by the number of defined daily doses (DDD) and defined course doses (DCD) per farm based on the values of Switzerland (DDDch/farm and DCDch/farm) and the European Medicine Agency (DDDvet/farm and DCDvet/farm). Each dataset was tested for normality by Shapiro-Wilk test and for all datasets the null hypothesis was rejected (each *P* < 0.001). So non-normal distributed data was concluded. By performing Kruskal-Wallis test for independent samples and *post hoc* pairwise analysis (Bonferroni correction) significant differences between finishing farm and farrow-to-finish-farm respectively, piglet-producing farm could be observed (each *P* < 0.001). No significant differences between farrow-to-finishing farm and piglet-producing farm could be observed (each *P* > 0.05).

Consideration of structure of the various farms pointed to a higher amount of calculated AMU per farm and per year on farrow-to-finishing farms and piglet-producing farms compared to finishing farms for all Swiss or European values of defined doses (*P* < 0.001) by Kruskal-Wallis-test and subsequent *post hoc* pairwise analysis (Table 4, Figure 3). In terms of calculated DDDch-numbers the median values were 4.40, 4.88, and 0.27 for farrow-to-finishing, piglet-producing and finishing farms, respectively. No significant difference between the farrow-to-finishing farms and the piglet-producers was observed for any of the used values.

**Table 4:** Median values of the defined daily doses (DDD) and defined course doses (DCD) based on the number of Switzerland (DDDch/farm and DCDch/farm) and the European Medicine Agency (DDDvet/farm and DCDvet/farm) for the different type of farms (farrow-to-finish farm, finishing farm and piglet-producing farm).

	**DDDch/farm**	**DDDvet/farm**	**DCDch/farm**	**DCDvet/farm**
1) Farrow-to-finish farm	4.40 (0.67–16.02)	3.63 (0.83–15.46)	1.43 (0.27–4.48)	0.98 (0.239–3.63)
2) Finishing farm	0.27^*^ (0–3.82)	0.26^*^ (0–2.75)	0.08^*^ (0–0.70)	0.077^*^ (0–0.50)
3) Piglet-producing farm	4.88 (0.96–12.45)	3.99 (1.04–12.04)	1.22 (0.29–4.61)	1.05 (0.26–2.65)

10 and 90% percentiles are given in brackets. Each dataset was tested for normality by Shapiro-Wilk tests and for all datasets the null hypothesis was rejected (each P < 0.001). So non-normal distributed data was concluded. By performing Kruskal-Wallis test for independent samples and post hoc pairwise analysis (Bonferroni correction) significant differences between finishing farm and farrow-to-finish-farm respectively, piglet-producing farm could be observed (each P < 0.001). No significant differences between farrow-to-finishing farm and piglet-producing farm could be observed (each P > 0.05).

* *P < 0.001 (to 1 and 3)*.

## Discussion

This study shows that although evaluating AMU for the pig sector at the farm level based either on Swiss or European defined doses leads to similar results with a positive correlated association, there were still deviations in detail, i.e., in the assessment of the different active substance classes, different administration routes and various age groups. A possible on farm AMU monitoring system will arrive at similar conclusions and farms with low or high AMU consumption will be similarly assessed using both methods. Since the Swiss definitions are based on individual national approvals in comparison to the average EMA definitions collected from nine countries, the Swiss definitions seem more robust for a national evaluation of active substance classes, administration routes and age groups.

The challenge of collecting adequate information on AMU in the field is well-known and described in the literature (15). Since the participation in the present study and supply of data was voluntary, some bias cannot be completely ruled out due to the fact that knowledge and motivation of farmers have an influence on AMU (24). We consider the coverage of the study population to be adequate for our study goals with 3.3% of all Swiss pig farms and 9.5% of all sows, and it allows to deduce that especially larger farms seemed to be more motivated to participate in the study.

Since the data underlying this study did not include a record about the length of pigs' stay in the farrowing unit, the nursery unit and the fattening unit, it is not feasible to make an exact evaluation of how many theoretical treatment days or treatments would be possible in the life span of a pig, as calculated by Timmermann et al. (25). However, the calculation behind the number of dosages on farm level is based on the population of animals present or produced during 1 year and this makes it comparable to other systems using defined doses to estimate AMU per farm in livestock (11–13).

Since the present study is based on calculations from prescribed amounts, the exact amounts of antimicrobials used by the farmer cannot be assessed and overdosing as well as underdosing could bias the results and the study only allows a statistical estimation of the probable AMU.

Another aspect of this study which is shown in Table 1 is the different evaluation of monitoring systems based either on the measurement of the amount of active ingredient or on the measurement of application equivalents such as defined doses: due to the lower standard weight of piglets, a considerable number of defined treatments can be performed with an amount of antimicrobial suitable for a single treatment of just one sow. As a consequence, the observed amount of active ingredients for e.g., piglets was low whereas the number of calculated doses was high. This is in line with prior studies (26) and EMA advice cautioning that differences in dosing between species and substances must be taken into account when using DDD and DCD values (19).

In general, a low value for a defined dose results in a higher number of calculated or estimated doses in a population (17). This explains some differences between the number of DDDch or DCDch on the one side and DDDvet and DCDvet on the other side. For example, macrolides showed a difference in calculated use depending on whether Swiss or European definitions were chosen. As a previous study showed, there are six Swiss premix products containing the macrolide tylosin with much lower defined daily and course doses compared to the values of the EMA (20), thus explaining the relatively high number of DDDch and DCDch in this category. This general understanding can also be used to explain the results in Table 1. All groups with a high ratio between the calculation based on Swiss or European definitions come by a frequent use with approvals whose DDDch and DCDch values differ strongly from the DDDvet and DCDvet values.

In accordance with a recently published study, the animal groups with the highest numbers of treatment days and total number of treatments observed were weaners (DDDch, DDDvet, and DCDvet) and piglets (DCDch) (27). These groups are most susceptible to bacterial infections and, at least for the weaners, frequent group therapies at weaning can be assumed, which is reflected in the high proportion of treatments with premixes, as described by other studies (28). This assumption is also underlined by the fact, that a longer Swiss treatment duration could be observed only for weaned piglets and for premixes and a relationship between both findings could be hypothesized. Thus, young age groups should already be considered in terms of resistance prevention and the use of group therapies by premixes in feed in these groups should be critically re-evaluated (29).

Furthermore, when calculating the number of DDDs, relatively high use could be observed for premixes and in contrast, a relatively high total number of course doses could be observed for injections in this study. This can be explained by the comparison of treatment durations between injections and premixes, since the number of calculated course doses decreases with the increase in treatment duration of the premixes. Previous publications confirmed the high proportion of premixes used in the pig sector in Switzerland (30, 31). An increased risk of development of resistance for specific active substances and bacteria is documented by this administration route (32). Thus, group therapies should be reduced to the necessary minimum.

A relatively high proportion of treatments with Highest Priority Critically Important Antimicrobials (HPCIAs) could be observed in this study (e.g., 44.8% of DDDvet's). These findings are comparable to results recently published from the EFFORT consortium (27), but varying from results of a previous study, where a lower AMU quantification of HPCIAs for pigs in Switzerland based on total amount of given active ingredient was observed (33). Due to the documented spread of resistance genes e.g., against fluoroquinolones in the pig sector (34), every use of these substances should be of concern and further research investigating restriction of indications and potential reductions in usage is needed.

The results from the different farm types show again that the younger age groups are most frequently treated. Both, farrow-to-finishing farm as well as piglet-producing farms in contrast to the finishing farms keep the high consumption age groups of piglets and weaners. This could explain the significant difference. Due to the small number of calculated defined doses of finisher pigs, no significant difference between farrow-to-finishing and piglet-producing farms was observed.

In order to gain a better understanding of the differences between these individual farms, further studies are needed to examine the role of the farmer (23) as well as AMU quantification and performance data (35).

## Conclusion

In summary, this study demonstrated a general association of the AMU systems at the farm level, nevertheless, differences were seen in detail according to whether the calculation was based on individual Swiss or average European values. The benefit of the European values for internationally comparative AMU monitoring is undisputed, but for a detailed evaluation, Swiss definitions could be more accurate as they are based on the specific approvals of the country. This must be considered in order to understand international AMU comparisons in the future. The study also highlighted the need to further evaluation for the use of HPCIAs.

## Data Availability

The datasets generated for this study are available on request to the corresponding author.

## Ethics Statement

Since this was only data that had no influence on the actual treatment of the animals, an animal welfare permit was not required. No manipulations or something similar were carried out on any animals.

## Author Contributions

DK, XS, CM, and TE contributed conception and design of the study. TE organized the database and performed the statistical analysis. CM is responsible for the correctness of the pharmacological formulations and the part in the manuscript about measurement methodologies. TE wrote the first draft of the manuscript. All authors contributed to manuscript revision, read, and approved the submitted version.

### Conflict of Interest Statement

The authors declare that the research was conducted in the absence of any commercial or financial relationships that could be construed as a potential conflict of interest. The handling Editor declared a past co-authorship with one of the authors CM.
