# Supplementary material for: Antimicrobial Drug Consumption on Swiss Pig Farms: A Comparison of Swiss and European Defined Daily and Course Doses in the Field
Source: Front Vet Sci. 2019 Jul 18;6:240. doi: 10.3389/fvets.2019.00240 (PMC6657740; doi:10.3389/fvets.2019.00240)
Supplement: Supplementary file 1 [file Data_Sheet_1.PDF]

Vetsuisse Faculty
Institute of Veterinary Pharmacology and Toxicology
Department for Farm Animals - Division of Swine Medicine

University of Zurich[UZH]

Establishment of the Defined Daily Dose Switzerland (DDDch) and Defined Course Dose Switzerland (DCDch) for all antimicrobial preparations approved for pigs in Switzerland

Decision Tree

In order to promote the responsible use of antibiotics, the use of antimicrobial agents is recorded and evaluated nationally and internationally. A recognised and widely used evaluation procedure consists of collecting defined daily doses and defined course doses. This method has been developed for human medicine and is based on defining the amount in mg for each active ingredient that is needed either daily or for an entire treatment of a standardized person lasting several days. Defined doses make it possible to estimate the potential treatment days or treatments (WHO, 2016) based on the total amount of active substances consumed. The consumption of antibiotics can be evaluated statistically in this way. Jensen et al. (2004) transferred this principle to veterinary medicine.
In 2016, the European Medicines Agency (EMA) published values for a defined daily dose (DDDvet) and a defined overall treatment dose (DCDvet) in the livestock sector (EMA, 2016). The values were calculated separately for pigs, cattle and poultry. DDDvet and DCDvet have been defined according to a defined procedure (EMA, 2015). First, data on the dosage of antimicrobial agents from nine member states were collected. For each active ingredient, the average daily and total treatment dosages were calculated from this data material and defined as DDDvet and DCDvet. The classification of the active substances was based on anatomical-technical-chemical congruencies (ATCvet Code) (WHO, 2017). In addition, the different application possibilities "Parenteral","Oral except Premix" and "Premix" for the pig area were taken into account and separate values for DDDvet and DCDvet were created. The value "Premix" summarizes the data of preparations that are approved for administration via feed or water and whose dosage and duration of treatment differs from the values for "oral except premix" approvals (tablets, boli etc.).

DDDvet and DCDvet enable a comparative evaluation of antibiotic use and form the basis of an international antibiotic monitoring system in farm animal medicine. However, the creation of average values from reports from different countries for the definition of DDDvet and DCDvet values has the disadvantage that national approvals and thus the dosages in an individual country can deviate considerably from these average values. Misinterpretations regarding the use of antibiotics are thus possible and the actual consumption is over- or underestimated.

Therefore, a national definition of daily and total treatment doses makes sense and has been implemented in several countries (DANMAP, 2016; NORM-VET, 2016; MARAN, 2017).

For these reasons, the values "Defined Daily Dose Switzerland (DDDch)" and "Defined Course Dose Switzerland (DCDch)" have been defined for all antimicrobial preparations approved in Switzerland for the treatment of pigs. The procedure was essentially based on

that of the EMA. Notwithstanding the principles of the EMA, DDDch and DCDch are not defined on the basis of the average dosages of each active substance, but for each preparation and the active substances contained therein individually.
The information required for the definition of the dosage of active substances in mg/kg and the intended duration of treatment shall, where possible, come from the Swiss Veterinary Medicinal Products Compendium (Tierarzneimittelkompendium TAK, 2017). DDDDch and DCDch always refer to the maximum dosage and duration of treatment for pigs. In contrast to the definition of other indices, for which the duration of the antibiotic effect of an active substance is used (effective days), the treatment days, i. e. the duration of the treatment in days, are decisive in this approach. This approach is also in line with the EMA's approach to defining DDDvet and DCDvet.

The decision tree shown here is intended to describe the solutions during the establishment of the DDDch and the DCDch. It will also show how the Swiss dosages (DDDch and DCDch) can be compared with the corresponding values of the EMA (DDDvet and DCDvet).
If possible, the decision tree assigns all recorded specimens to a category. The categories are explained and summarized in figure 1. Next to the decision tree, there is a commented table of the collected specimens (Table 1.). All antimicrobial preparations approved for pigs in Switzerland on 1 May 2017 were included.

Category 1 (DDDch / DCDch values and DDDvet / DCDvet values clearly defined):
Preparations for which detailed information on active substances contained in the TAK of Switzerland and their dosage in mg/kg as well as a clear duration of treatment are given. In these cases, the definition of DDDch and DCDch follows the concept of EMA very closely. In addition, there are clear DDDvet and DCDvet values of the EMA for the active substances contained in the preparation.

Category 2 (special explanations for DDDch and DCDch values):
Preparations for which the DDDch and DCDch values cannot be clearly defined according to TAK for various reasons.

Category 2.1 (no exact treatment duration in TAK):
In the case of preparations for which no exact treatment duration was stored in the TAK, it is not possible to define the DCDch unambiguously:
The first option for these preparations was the treatment duration of a comparable Swiss preparation that had the same composition of active ingredients for the same application form. If no such preparation was available, the second option was the duration of treatment used by the EMA (EMA, 2016). This was calculated using the quotient from DCDvet and DDDvet. In this way, it was possible to assign a treatment duration to all approved preparations in Switzerland and to define the DCDch.

Category 2.2 (different data on concentration in TAK):
Preparations which contain identical active substances but whose composition differs in mg/kg product (concentration):
The dosages of these preparations deposited in the TAK differ from each other because they apply to different indications. This would also make the values DDDch and DCDch unequal. For practical reasons, the DDDch and DCDch values of these preparations have been standardized. Rare indications such as rare diseases or disease complexes or diseases that are not to be treated under animal disease legislation have been neglected.

For example, the active ingredient enrofloxacin is available in differently concentrated preparations. However, only part of these preparations are approved for the indication salmonellosis. Salmonellosis would be the indication with the highest dosage and the longest duration of treatment and thus, in principle, the indication to be chosen for the establishment of DDDch and DCDch. However, this would result in several DDDch and DCDch values for preparations containing the same active ingredient (enrofloxacin). Therefore, the rare indication salmonellosis was not taken into account at this point and the DDDch and DCDch were defined deviating from the maximum dosage and duration of treatment.
The neglected and selected indications for the determination of DDDch and DCDch are given in the commentary.

Category 2.3: (various indications in the TAK):
Preparations in which different dosages were found in TAK depending on the indication or form of treatment (therapy or prophylaxis):
In this case, the same procedure as described under point 2.2 was followed and rare or irrelevant indications for Switzerland were excluded, thus defining the dosage for the respective preparations on the basis of a common indication. The choice of indication and thus the definition of DDDch and DCDch was strictly based on practical and pharmacological aspects. Preparations for which this procedure has been followed are indicated in the commentary together with the selected indication.

Category 2.4: (changed daily dosages during treatment):
Preparations in which the daily dosage changes during the course of treatment according to the TAK:
In these cases, the daily dosage of the DDDDch was calculated in such a way that the total treatment dose (DCDch) was divided by the treatment days. Thus, the DDDDch reflects an average of the individual daily dosages. These preparations are also commented accordingly in the table for better understanding.

Category 3 (special explanations for DDDvet and DCDvet values):

Category 3.1 (no unique DDDvet and DCDvet values):
In its procedure, the EMA has stipulated that only additional DDDvet and DCDvet values for active substances in combination should be created if the combination results in a different dosage. For example, the synergistic effect of sulfonamides and trimethoprim was taken into account, as well as the highly deviating dosage of lincosamides and spectinomycin in combination. The remaining reported dosages of active substances in combination preparations were recorded and included in the DDDvet and DCDvet values of the corresponding non-combined active substances. Additional DDDvet and DCDvet values for combinations were not calculated.
The same applies to the application form "Premix", which is not specifically indicated. Here, only an additional value for "Premix" was created if it was reported by one of the nine member states.
The European Surveillance of Veterinary Antimicrobial Consumption (ESVAC), which has created the procedure and defined the DDDvet and DCDvet values, points out that in cases where there are no corresponding values for combinations or combinations, respectively. DDDvet and DCDvet values for non-combined active ingredients or "oral except premix" (EMA, 2015; EMA, 2016).

Category 3.2 (DDDvet and DCDvet values not defined):
There are several reasons for this:

Category 3.2.1: No DDDvet and DCDvet values have been created for topical preparations, as no exact dosage in mg/kg is used here. Based on the information provided by the TAK, values were defined for the Swiss values with the help of pharmacological expertise, so that a DDDch and DCDch are available.

Category 3.2.2: The preparations Draxxin® (Tulathromycin) and Zuprevo® (Tildipirosine) have not yet been listed by the EMA because they were not yet definitively defined as "long acting" preparations. For Switzerland, the values were determined on the basis of the TAK and equated to DDDch and DCDch.

Category 3.2.3: Preparations containing Sulfathiazole have not yet been considered by the EMA as they are not used in any of the 9 Member States. The Swiss values DDDch and DCDch were created using TAK.

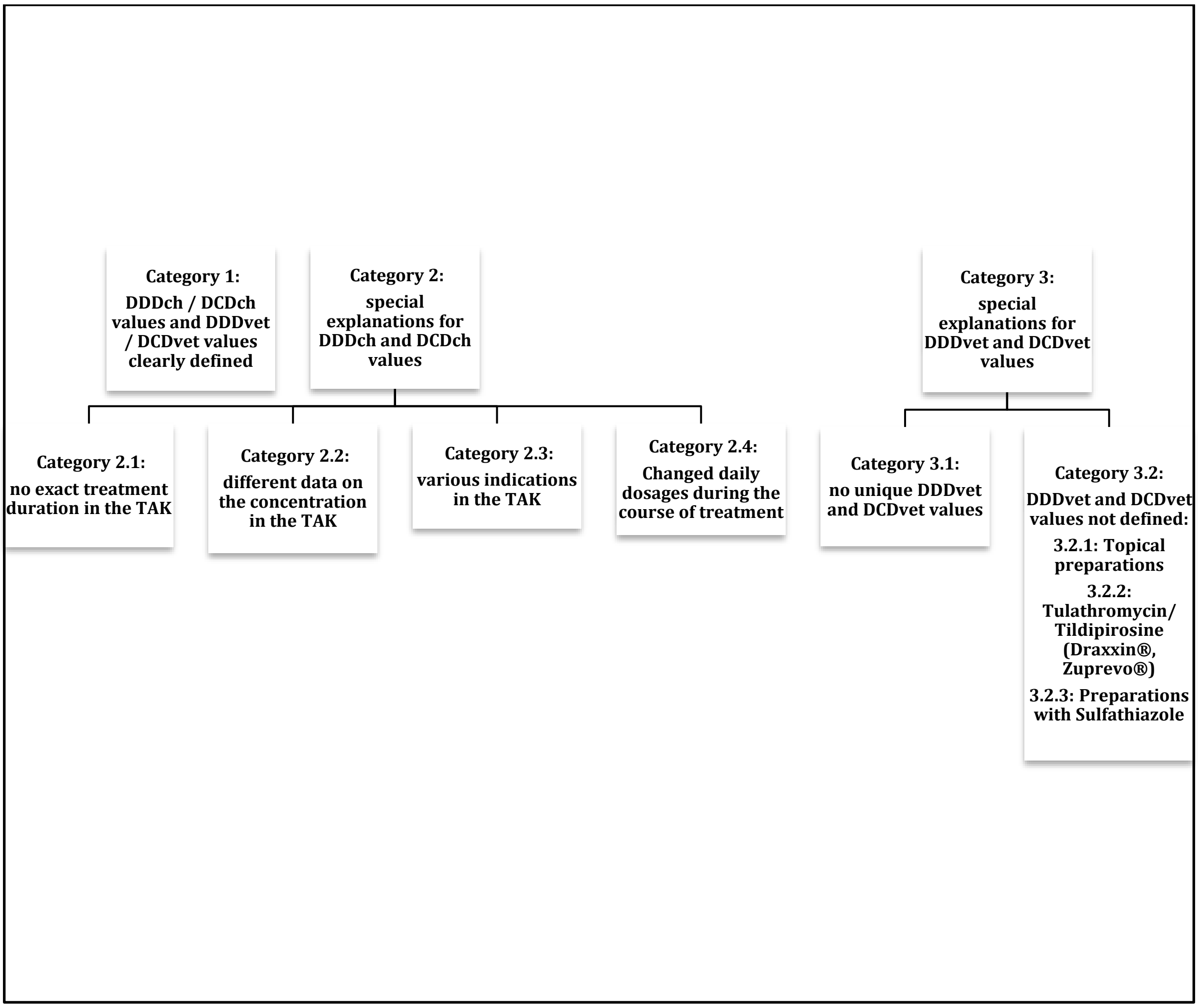

Figure 1: Overview of the decision tree, (TAK= Swiss Veterinary Medicines Compendium)

Table 1: Classification of all antimicrobial active substances approved for pigs in Switzerland

Category	Drug	Comment
**Category 1**		**DDDch / DCDch values and DDDvet / DCDvet values clearly defined:**
	Advocid 2,5% ad us. vet., Injektionslösung	
	Alamycin LA ad us. vet., Injektionslösung	
	Albipen LA ad us. vet., Injektionssuspension	
	Amoxan 70 ad us. vet., Arzneimittelvormischung	
	Aurofac 100 Granular ad us. vet., Arzneimittelvormischung	
	B-COL 500 ad us. vet., Arzneimittelvormischung	
	Baxyl LA 200 ad us. vet., Injektionslösung	
	Baytril 0,5% ad us. vet., orale Lösung	
	Baytril MAX 100 mg/ml ad us. vet., Injektionslösung	
	Betamox LA ad us. vet., Injektionssuspension	
	Boflox 10% ad us. vet., Injektionslösung	
	Cefenil ad us. vet., Injektionslösung	
	Ceftiocyl ad us. vet., Injektionssuspension	
	Clamoxyl ad us. vet., ölige Suspension	
	Colfen 300 mg/ml ad us. vet. [V], Injektionslösung	
	Cyclosol LA ad us. vet., Injektionslösung	
	Doxivit ad us. vet., Arzneimittelvormischung	
	Duphamox L.A. ad us. vet., Injektionssuspension	
	Eficur RTU ad us. vet., Injektionssuspension	
	Emorex N Berna ad us. vet., Granulat	
	Emorex N Berna ad us. vet., Tabletten	
	Engemycin 10% ad us. vet.	
	Excenel 1 g ad us. vet., Injektionslösung	
	Excenel Fluid ad us. vet., Injektionssuspension	
	Forcyl 160 mg/ml ad us. vet., Injektionslösung	
	Gentamycin 4% Virbac ad us. vet. [A], Injektionslösung	
	Ilcocillin P300 ad us. vet., Injektionssuspension	
	Linco-Spectin ad us. vet., Injektionslösung	
	Linco-Spectin Premix ad us. vet., Arzneimittelvormischung	
	Longamox ad us. vet., Injektionssuspension	
	Marbocyl 10% ad us. vet., Injektionslösung	
	Naxcel 100 mg/ml Schwein ad us. vet., Injektionssuspension	
	Norfenicol 300 mg/ml ad us. vet., Injektionslösung	
	Nuflor 300 mg/ml ad us. vet., Injektionslösung	

	Paracillin SP ad us. vet., Arzneimittelvormischung (lösliches Pulver)	
	Primadox 50 ad us. vet., Arzneimittelvormischung<br>Procacillin ad us. vet., Injektionssuspension	
	Pulmotil AC ad us. vet., Arzneimittelvormischung	
	Shotaflor 300 mg/ml ad us. vet., Injektionslösung	
	Stabox 10% ad us. vet., Arzneimittelvormischung	
	Stabox 50% ad us. vet., Arzneimittelvormischung	
	Synulox Suspension ad us. vet., Injektionssuspension	The contained clavulanic acid was not included as active ingredient because it has no significant antimicrobial effect.
	Terralon 20% LA ad us. vet., Injektionslösung	
	Tylan 200 ad us. vet., Injektionslösung	
	ufamed Colistin 500 ad us. vet., Arzneimittelvormischung	
	Vesuprim ad us. vet., wässrige Injektionslösung	
	VITAL COLISTIN 125 ad us. vet., Arzneimittelvormischung	
	VITAL COLISTIN 2500 ad us. vet., Arzneimittelvormischung	
	VITAL COLISTIN 500 ad us. vet., Arzneimittelvormischung	
**Categpry 2:**		**Explanations of the values of Switzerland (DDDch + DCDch):**
**Category 2.1.**	Ampitab ad us. vet., Tabletten	The TAK did not specify a duration of treatment. There is also no other preparation with identical active ingredients and composition. For this reason, the EMA was used for the number of treatment days and the rounded value was taken over.
	Borgal 24% ad us. vet., Injektionslösung	The TAK did not specify a duration of treatment. treatment duration of comparable preparations (with identical active substances and the same composition); defined here on the basis of identical active substance groups, orientates itself on: Vesuprim ad us. vet, aqueous injection solution
	Duphacycline L.A. ad us. vet., Injektionslösung	The TAK did not specify a duration of treatment. Duration of treatment of comparable preparations (with identical active substances and the same composition) taken over
	Pargenta-50 ad us. vet., Injektionslösung	The TAK did not specify a duration of treatment. There is also no other preparation with identical active ingredients and composition. For this reason, the EMA was used for the number of treatment days and the rounded value was taken over.
	Pharmasin 200 ad us. vet., Injektionslösung	The TAK did not specify a duration of treatment. Duration of treatment of comparable preparations (with identical active substances and the same composition) taken over
	Rota-TS ad us. vet., Pulver	The TAK did not specify a duration of treatment. There is also no other preparation

		with identical active ingredients and composition. For this reason, the EMA was used for the number of treatment days and the rounded value was taken over.
	Streptopenicillin 45 mega ad us. vet., Injektionssuspension *	The TAK did not specify a duration of treatment. Duration of treatment of comparable preparations (with identical active substances and same composition) adopted; *Preparation also appears in category 3.1
	Trimethazol ad us. vet., Injektionslösung	The TAK did not specify a duration of treatment. treatment duration of comparable preparations (with identical active substances and the same composition); defined here on the basis of identical active substance groups, orientates itself on: Vesuprim ad us. vet, aqueous injection solution
	Tylucyl 200 ad us. vet., Injektionslösung	The TAK did not specify a duration of treatment. Duration of treatment of comparable preparations (with identical active substances and the same composition) taken over
	Vetagent ad us. vet., Injektionslösung	The TAK did not specify a duration of treatment. There is also no other preparation with identical active ingredients and composition. For this reason, the EMA was used for the number of treatment days and the rounded value was taken over.
	Westocillin ad us. vet., Injektionssuspension *	The TAK did not specify a duration of treatment. Duration of treatment of comparable preparations (with identical active substances and same composition) adopted; *Preparation also appears in category 3.1
**Category 2.2**	Baytril 10% ad us. vet., Injektionslösung	Treatment duration between the preparations unified, salmonellosis and complicated respiratory tract infection not considered as indications, Postpartum Dysgalactia Syndrome selected as indication
	Baytril 2,5% ad us. vet., Injektionslösung	Treatment duration between the preparations unified, salmonellosis and complicated respiratory tract infection not considered as indications, Postpartum Dysgalactia Syndrome selected as indication
	Baytril 5% ad us. vet., Injektionslösung	Treatment duration between the preparations unified, salmonellosis and complicated respiratory tract infection not considered as indications, Postpartum Dysgalactia Syndrome selected as indication
	Enrotron 10% ad us. vet., Injektionslösung	Treatment duration between the preparations unified, salmonellosis and complicated respiratory tract infection not considered as indications, Postpartum Dysgalactia Syndrome selected as indication
	Enrotron 2,5% ad us. vet., Injektionslösung	Treatment duration between the preparations unified, salmonellosis and complicated respiratory tract infection not considered as indications, Postpartum Dysgalactia Syndrome selected as indication
	Enrotron 5% ad us. vet., Injektionslösung	Treatment duration between the preparations unified, salmonellosis and complicated respiratory tract infection not considered as indications, Postpartum Dysgalactia Syndrome

		selected as indication
	Marbocyl 10% ad us. vet., Injektionslösung	Treatment duration between preparations standardized, Enzootic pneumonia (EP), Actinobacillus pleuropneumonia (APP) and progressive Rhinitis atrophicans (pRA) not considered as indication, Postpartum Dysgalactia Syndrome (PPDS) selected as indication
	Marbocyl 2% ad us. vet., Injektionslösung	Treatment duration between preparations standardized, Enzootic pneumonia (EP), Actinobacillus pleuropneumonia (APP) and progressive Rhinitis atrophicans (pRA) not considered as indication, Postpartum Dysgalactia Syndrome (PPDS) selected as indication
	Marbox 100 ad us. vet., Injektionslösung	Treatment duration between preparations standardized, Enzootic pneumonia (EP), Actinobacillus pleuropneumonia (APP) and progressive Rhinitis atrophicans (pRA) not considered as indication, Postpartum Dysgalactia Syndrome (PPDS) selected as indication
	Marfloquin 10% ad us. vet., Injektionslösung	Treatment duration between preparations standardized, Enzootic pneumonia (EP), Actinobacillus pleuropneumonia (APP) and progressive Rhinitis atrophicans (pRA) not considered as indication, Postpartum Dysgalactia Syndrome (PPDS) selected as indication
	Marfloquin 2% ad us. vet., Injektionslösung	Treatment duration between preparations standardized, Enzootic pneumonia (EP), Actinobacillus pleuropneumonia (APP) and progressive Rhinitis atrophicans (pRA) not considered as indication, Postpartum Dysgalactia Syndrome (PPDS) selected as indication
	Powerflox 100 ad us. vet., Injektionslösung	Standardized treatment duration between preparations, Salmonellosis not taken into account as indication, "basic dosage" selected as indication
	Powerflox 50 ad us. vet., Injektionslösung	Standardized treatment duration between preparations, Salmonellosis not taken into account as indication, "basic dosage" selected as indication
**Category 2.3**	Cobactan 2.5% ad us. vet., Injektionssuspension	For Switzerland, the indications for piglets were not taken into account and the dosage for "bacterial respiratory infections" in pigs was chosen
	Colivet Quick pump ad us. vet., orale Suspension	In the TAK, a doubling of the dosage would be possible in "serious cases". This was not taken into account
	Denagard 100 ad us. vet., Injektionslösung	For the Swiss values, the indication Enzootic Pneumonia (EP) was not taken into account and the next higher dosages for the indication Mycoplasma Arthritis were chosen
	Denagard Premix 10% ad us. vet., Arzneimittelvormischung	For Switzerland, the dosages for Porcine Proliferative Enteropathy (Ileitis) were chosen
	Econor 10% ad us. vet. [A], Arzneimittelvormischung	For Switzerland, the dosages for Porcine Proliferative Enteropathy (Ileitis) were chosen
	Stabox 5% ad us. vet.,	Dosage for Streptococcus suis is not

	Arzneimittelvormischung	considered as an indication. Dosage for "other amoxicillin-sensitive pathogens" selected
	Tylan soluble ad us. vet., Arzneimittelvormischung	For Switzerland, the dosages for the "short-term treatment" of ileitis caused by Lawsonia intracellularis were chosen
**Category 2.4**	Enteran ad us. vet., Pulver *	The daily dosage indicated in the TAK changes during treatment, so that the Swiss value DDDch is to be understood as the average of the individual daily dosages. preparation also appears in category 3.2.3
	Tandozin ad us. vet., Injektionslösung *	The daily dosage indicated in the TAK changes during treatment, so that the Swiss value DDDch is to be understood as the average of the individual daily dosages; *Preparation also appears in category 3.1
**Category 3:**		**Explanation of the values of the EMA (DDDvet + DCDvet):**
**Category 3.1.**	B-TS Duo 75 ad us. vet., Arzneimittelvormischung	For sulfadimidine, the value for "Oral except premix" was selected for the combination of sulfadimidine and trimethoprim because no "premix" value was reported
	CAS 45 K ad us. vet., Arzneimittelvormischung	Dosages for "Premix" mono-preparations are selected, as no additional combination dosages have been published for the active substances
	CAS 45 S ad us. vet., Arzneimittelvormischung	Dosages for "Premix" mono-preparations are selected, as no additional combination dosages have been published for the active substances
	Cobiotic N ad us. vet., wässrige Suspension	Dosages for "parenteral" monopreparations are selected because no additional combination dosages have been published for the active substances contained in them
	Duplocillin LA ad us. vet., Injektionssuspension	Dosages for parenteral mono-preparations were selected, as no additional combination dosage was published for this active ingredient. The Swiss values were defined with the help of TAK's and pharmacological expertise
	Ilcocillin PS200/200 ad us. vet., Injektionssuspension	Dosages for "parenteral" monopreparations are selected because no additional combination dosages have been published for the active substances contained in them
	Norocillin LA ad us. vet., Injektionssuspension	Dosages for parenteral mono-preparations were selected, as no additional combination dosage was published for this active ingredient. The Swiss values were defined with the help of TAK's and pharmacological expertise
	Pen-Strep 20/20 ad us. vet., Injektionssuspension	Dosages for "parenteral" monopreparations are selected because no additional combination dosages have been published for the active substances contained in them
	SK-40 ad us. vet., Arzneimittelvormischung	Dosages for "Premix" mono-preparations are selected, as no additional combination dosages have been published for the active substances
	SK-60 ad us. vet., Arzneimittelvormischung	Dosages for "Premix" mono-preparations are selected, as no additional combination dosages have been published for the active substances
	Streptopenicillin 45 mega ad us. vet., Injektionssuspension *	Dosages for parenteral mono compounds are selected because no additional combination

		dosages have been published for the active ingredients; *Preparation also appears in category 2.1.
	Tandozin ad us. vet., Injektionslösung *	Dosages for parenteral mono preparations selected, as no additional combination dosages have been published for the active substances contained; *Preparation also appears in category 2.4
	Vital CST-222 ad us. vet., Arzneimittelvormischung	Dosages for "Premix" mono-preparations are selected, as no additional combination dosages have been published for the active substances
	Vital CST-222 L ad us. vet., Arzneimittelvormischung	Dosages for "Premix" mono-preparations are selected, as no additional combination dosages have been published for the active substances
	Westocillin ad us. vet., Injektionssuspension *	Dosages for parenteral mono compounds are selected because no additional combination dosages have been published for the active ingredients; *Preparation also appears in category 2.1.
**Category 3.2:**		**No DDDvet and DCDvet value available:**
**Category 3.2.1**	Alamycin Spray ad us. vet.	Topical active ingredients were not considered by the EMA. The Swiss dosage was defined with the help of TAK's and pharmacological expertise
	Chlor-Tetracyclin-Spray Stricker ad us. vet.	Topical active ingredients were not considered by the EMA. The Swiss dosage was defined with the help of TAK's and pharmacological expertise
	Cyclospray ad us. vet., Spray	Topical active ingredients were not considered by the EMA. The Swiss dosage was defined with the help of TAK's and pharmacological expertise
	Engemycin Spray ad us. vet.	Topical active ingredients were not considered by the EMA. The Swiss dosage was defined with the help of TAK's and pharmacological expertise
**Category 3.2.2**	Draxxin 10% ad us. vet., Injektionslösung	The active ingredient Tulathromycin LA has not yet been taken into account by the EMA. For Switzerland, the values were determined on the basis of the TAK
	Zuprevo® 40 mg/ml ad us. vet.[A], Injektionslösung	The active ingredient Tildipirosine LA has not yet been taken into account by the EMA. For Switzerland, the values were determined on the basis of the TAK.
**Category 3.2.3**	B-TS Trio ad us. vet., Arzneimittelvormischung	Contains Sulfathiazol, an active ingredient that has not been taken into account by the EMA
	UFA 902 DUO ad us. vet., Arzneimittelvormischung	Contains Sulfathiazol, an active ingredient that has not been taken into account by the EMA
	Vital TSS 480 ad us. vet., Arzneimittelvormischung	Contains Sulfathiazol, an active ingredient that has not been taken into account by the EMA
	Vital TSS 96 ad us. vet., Arzneimittelvormischung	Contains Sulfathiazol, an active ingredient that has not been taken into account by the EMA
	Enteran ad us. vet., Pulver *	Contains Sulfathiazol, an active ingredient that has not been taken into account by the EMA *Preparation also appears in category 2.4.

Table 2: All antimicrobial approvals authorised for use in pigs in Switzerland, including their daily and treatment dosages

Approval	Active ingredients	ATCvet-Code	Administration route	DDDch/kg	Treatment duration	DCDch/kg
Advocid 2,5% ad us. vet., Injektionslösung	Danofloxacin	QJ01MA92	Injection	1.25	3	3.75
Alamycin LA ad us. vet., Injektionslösung	Oxytetracyclin	QJ01AA06	Injection	20	2	40
Alamycin Spray ad us. vet.	Oxytetracyclin	QD06AA03	Topical	12.5	3	37.5
Albipen LA ad us. vet., Injektionssuspension	Ampicillin	QJ01CA01	Injection	25	2	50
Amoxan 70 ad us. vet., Arzneimittelvormischung	Amoxicillin	QJ01CA04	Premix	21	5	105
Ampitab ad us. vet., Tabletten	Ampicillin	QJ01CA01	Oral	12	4	48
Aurofac 100 Granular ad us. vet., Arzneimittelvormischung	Chlortetracyclin	QJ01AA03	Premix	30	14	420
B-COL 500 ad us. vet., Arzneimittelvormischung	Colistin	QA07AA10	Premix	6	10	60
B-TS Duo 75 ad us. vet., Arzneimittelvormischung	Sulfadimidin_TMP	QJ01EW03	Premix	25	7	175
B-TS Duo 75 ad us. vet., Arzneimittelvormischung	Trimethoprim_sulfa	QJ01EW03	Premix	5	7	35
B-TS Trio ad us. vet., Arzneimittelvormischung	Sulfadimidin_TMP	QJ01EW30	Premix	20	10	200
B-TS Trio ad us. vet., Arzneimittelvormischung	Sulfathiazol_TMP	QJ01EW30	Premix	20	10	200
B-TS Trio ad us. vet., Arzneimittelvormischung	Trimethoprim_sulfa	QJ01EW30	Premix	8	10	80
Baxyl LA 200 ad us. vet., Injektionslösung	Oxytetracyclin	QJ01AA06	Injection	20	2	40
Baytril 0,5% ad us. vet., orale Lösung	Enrofloxacin	QJ01MA90	Oral	1.67	3	5
Baytril 10% ad us. vet., Injektionslösung	Enrofloxacin	QJ01MA90	Injection	2.5	3	7.5
Baytril 2,5% ad us. vet., Injektionslösung	Enrofloxacin	QJ01MA90	Injection	2.5	3	7.5
Baytril 5% ad us. vet., Injektionslösung	Enrofloxacin	QJ01MA90	Injection	2.5	3	7.5
Baytril MAX 100 mg/ml ad us. vet., Injektionslösung	Enrofloxacin	QJ01MA90	Injection	7.5	1	7.5
Betamox LA ad us. vet., Injektionssuspension	Amoxicillin	QJ01CA04	Injection	15	2	30
Boflox 10% ad us. vet., Injektionslösung	Marbofloxacin	QJ01MA93	Injection	2	3	6
Borgal 24% ad us. vet., Injektionslösung	Sulfadoxinum_TMP	QJ01EW13	Injection	12	4	48
Borgal 24% ad us. vet., Injektionslösung	Trimethoprim_sulfa	QJ01EW13	Injection	2.4	4	9.6

CAS 45 K ad us. vet., Arzneimittelvormischung	Chlortetracyclin	QJ01RA02	Premix	22.5	10	225
CAS 45 K ad us. vet., Arzneimittelvormischung	Sulfadimidin	QJ01RA02	Premix	36	10	360
CAS 45 K ad us. vet., Arzneimittelvormischung	Tylosin	QJ01RA02	Premix	3.6	10	36
CAS 45 S ad us. vet., Arzneimittelvormischung	Chlortetracyclin	QJ01RA02	Premix	21	10	210
CAS 45 S ad us. vet., Arzneimittelvormischung	Sulfadimidin	QJ01RA02	Premix	42	10	420
CAS 45 S ad us. vet., Arzneimittelvormischung	Tylosin	QJ01RA02	Premix	3.6	10	36
Cefenil ad us. vet., Injektionslösung	Ceftiofur	QJ01DD90	Injection	3	3	9
Ceftiocyl ad us. vet., Injektionssuspension	Ceftiofur	QJ01DD90	Injection	3	3	9
Chlor-Tetracyclin-Spray Stricker ad us. vet.	Chlortetracyclin	QD06AA02	Topical	12.5	3	37.5
Clamoxyl ad us. vet., ölige Suspension	Amoxicillin	QJ01CA04	Injection	7	5	35
Cobactan 2.5% ad us. vet., Injektionssuspension	Cefquinom	QJ01DE90	Injection	2	3	6
Cobiotic N ad us. vet., wässrige Suspension	Benzylpenicillinum procainum	QJ01RA01	Injection	20	2	40
Cobiotic N ad us. vet., wässrige Suspension	Dihydrostreptomycin	QJ01RA01	Injection	25	2	50
Colfen 300 mg/ml ad us. vet. [V], Injektionslösung	Florfenicol	QJ01BA90	Injection	15	2	30
Colivet Quick pump ad us. vet., orale Suspension	Colistin	QA07AA10	Oral	6.4	5	32
Cyclosol LA ad us. vet., Injektionslösung	Oxytetracyclin	QJ01AA06	Injection	20	2	40
Cyclospray ad us. vet., Spray	Chlortetracyclin	QD06AA02	Topical	7.61	3	22.82
Denagard 100 ad us. vet., Injektionslösung	Tiamulin	QJ01XQ01	Injection	12.5	3	37.5
Denagard Premix 10% ad us. vet., Arzneimittelvormischung	Tiamulin	QJ01XQ01	Premix	7.5	14	105
Doxivit ad us. vet., Arzneimittelvormischung	Doxycyclin	QJ01AA02	Premix	12.5	8	100
Draxxin 10% ad us. vet., Injektionslösung	Tulathromycin_LA	QJ01FA94	Injection	2.5	1	2.5
Duphacycline L.A. ad us. vet., Injektionslösung	Oxytetracyclin	QJ01AA06	Injection	20	2	40
Duphamox L.A. ad us. vet., Injektionssuspension	Amoxicillin	QJ01CA04	Injection	15	2	30
Duplocillin LA ad us. vet., Injektionssuspension	Benzylpenicillinum benzathinum	QJ01CE30	Injection	3.6	1	3.6
Duplocillin LA ad us. vet., Injektionssuspension	Benzylpenicillinum procainum	QJ01CE30	Injection	3.6	1	3.6
Econor 10% ad us. vet. [A], Arzneimittelvormischung	Valnemulin	QJ01XQ02	Premix	4	14	56

Eficur RTU ad us. vet., Injektionssuspension	Ceftiofur	QJ01DD90	Injection	3	3	9
Emorex N Berna ad us. vet., Granulat	Neomycin	QA07AA01	Oral	46.67	4	186.67
Emorex N Berna ad us. vet., Tabletten	Neomycin	QA07AA01	Oral	70	4	280
Engemycin 10% ad us. vet.	Oxytetracyclin	QJ01AA06	Injection	20	2	40
Engemycin Spray ad us. vet.	Oxytetracyclin	QD06AA03	Topical	12.5	3	37.5
Enrotron 10% ad us. vet., Injektionslösung	Enrofloxacin	QJ01MA90	Injection	2.5	3	7.5
Enrotron 2,5% ad us. vet., Injektionslösung	Enrofloxacin	QJ01MA90	Injection	2.5	3	7.5
Enrotron 5% ad us. vet., Injektionslösung	Enrofloxacin	QJ01MA90	Injection	2.5	3	7.5
Enteran ad us. vet., Pulver	Neomycin	QA07AA51	Oral	50	3	150
Enteran ad us. vet., Pulver	Sulfathiazol	QA07AA51	Oral	416.67	3	1250
Excenel 1 g ad us. vet., Injektionslösung	Ceftiofur	QJ01DD90	Injection	3	3	9
Excenel Fluid ad us. vet., Injektionssuspension	Ceftiofur	QJ01DD90	Injection	3	3	9
Forcyl 160 mg/ml ad us. vet., Injektionslösung	Marbofloxacin	QJ01MA93	Injection	8	1	8
Gentamycin 4% Virbac ad us. vet. [A], Injektionlösung	Gentamicin	QJ01GB03	Injection	5	1	5
Ilcocillin P300 ad us. vet., Injektionssuspension	Benzylpenicillinum procainum	QJ01CE09	Injection	10	5	50
Ilcocillin PS200/200 ad us. vet., Injektionssuspension	Benzylpenicillinum procainum	QJ01RA01	Injection	8	3	24
Ilcocillin PS200/200 ad us. vet., Injektionssuspension	Dihydrostreptomycin	QJ01RA01	Injection	8	3	24
Linco-Spectin ad us. vet., Injektionslösung	Lincomycin	QJ01FF52	Injection	5	7	35
Linco-Spectin ad us. vet., Injektionslösung	Spectinomycin	QJ01FF52	Injection	10	7	70
Linco-Spectin Premix ad us. vet., Arzneimittelvormischung	Lincomycin	QJ01FF52	Premix	2.2	21	46.2
Linco-Spectin Premix ad us. vet., Arzneimittelvormischung	Spectinomycin	QJ01FF52	Premix	2.2	21	46.2
Longamox ad us. vet., Injektionssuspension	Amoxicillin	QJ01CA04	Injection	15	2	30
Marbocyl 10% ad us. vet., Injektionslösung	Marbofloxacin	QJ01MA93	Injection	2	3	6
Marbocyl 2% ad us. vet., Injektionslösung	Marbofloxacin	QJ01MA93	Injection	2	3	6
Marfloquin 10% ad us. vet., Injektionslösung	Marbofloxacin	QJ01MA93	Injection	2	3	6
Marbox 100 ad us. vet., Injektionslösung	Marbofloxacin	QJ01MA93	Injection	2	3	6

Marfloquin 2% ad us. vet., Injektionslösung	Marbofloxacin	QJ01MA93	Injection	2	3	6
Naxcel 100 mg/ml Schwein ad us. vet., Injektionssuspension	Ceftiofur_LA	QJ01DD90	Injection	0.8	6.25	5
Norfenicol 300 mg/ml ad us. vet., Injektionslösung	Florfenicol	QJ01BA90	Injection	15	2	30
Norocillin LA ad us. vet., Injektionssuspension	Benzylpenicillinum benzathinum	QJ01CE30	Injection	3.6	1	3.6
Norocillin LA ad us. vet., Injektionssuspension	Benzylpenicillinum procainum	QJ01CE30	Injection	3.6	1	3.6
Nuflor 300 mg/ml ad us. vet., Injektionslösung	Florfenicol	QJ01BA90	Injection	15	2	30
Paracillin SP ad us. vet., Arzneimittelvormischung (lösliches Pulver)	Amoxicillin	QJ01CA04	Premix	14	5	70
Pargenta-50 ad us. vet., Injektionslösung	Gentamicin	QJ01GB03	Injection	4	4	16
Pen-Strep 20/20 ad us. vet., Injektionssuspension	Benzylpenicillinum procainum	QJ01RA01	Injection	10	3	30
Pen-Strep 20/20 ad us. vet., Injektionssuspension	Dihydrostreptomycin	QJ01RA01	Injection	10	3	30
Pharmasin 200 ad us. vet., Injektionslösung	Tylosin	QJ01FA90	Injection	10	3	30
Powerflox 100 ad us. vet., Injektionslösung	Enrofloxacin	QJ01MA90	Injection	2.5	3	7.5
Powerflox 50 ad us. vet., Injektionslösung	Enrofloxacin	QJ01MA90	Injection	2.5	3	7.5
Primadox 50 ad us. vet., Arzneimittelvormischung	Doxycyclin	QJ01AA02	Premix	12.5	8	100
Procacillin ad us. vet., Injektionssuspension	Benzylpenicillinum procainum	QJ01CE09	Injection	15	5	75
Pulmotil AC ad us. vet., Arzneimittelvormischung	Tilmicosin	QJ01FA91	Premix	20	5	100
Rota-TS ad us. vet., Pulver	Trimethoprim_sulfa	QJ01EW03	Oral	3.4	5	17
Rota-TS ad us. vet., Pulver	Sulfadimidin_TMP	QJ01EW03	Oral	17	5	85
Shotaflor 300 mg/ml ad us. vet., Injektionslösung	Florfenicol	QJ01BA90	Injection	15	2	30
SK-40 ad us. vet., Arzneimittelvormischung	Chlortetracyclin	QJ01RA02	Premix	20	7	140
SK-40 ad us. vet., Arzneimittelvormischung	Sulfadimidin	QJ01RA02	Premix	40	7	280
SK-40 ad us. vet., Arzneimittelvormischung	Tylosin	QJ01RA02	Premix	4	7	28
SK-60 ad us. vet., Arzneimittelvormischung	Spiramycin	QJ01RA90	Premix	18	10	180
SK-60 ad us. vet., Arzneimittelvormischung	Chlortetracyclin	QJ01RA90	Premix	24	10	240
Stabox 10% ad us. vet., Arzneimittelvormischung	Amoxicillin	QJ01CA04	Premix	21	5	105
Stabox 5% ad us. vet., Arzneimittelvormischung	Amoxicillin	QJ01CA04	Premix	20	5	100

Stabox 50% ad us. vet., Arzneimittelvormischung	Amoxicillin	QJ01CA04	Premix	20	5	100
Streptopenicillin 45 mega ad us. vet., Injektionssuspension	Benzylpenicillinum procainum	QJ01RA01	Injection	20	2	40
Streptopenicillin 45 mega ad us. vet., Injektionssuspension	Dihydrostreptomycin	QJ01RA01	Injection	25	2	50
Synulox Suspension ad us. vet., Injektionssuspension	Amoxicillin	QJ01CR02	Injection	7	5	35
Tandozin ad us. vet., Injektionslösung	Sulfadimidin	QJ01EQ30	Injection	6.67	3	20
Tandozin ad us. vet., Injektionslösung	Sulfamethoxypyridazi num	QJ01EQ30	Injection	13.32	3	40
Terralon 20% LA ad us. vet., Injektionslösung	Oxytetracyclin	QJ01AA06	Injection	20	2	40
Trimethazol ad us. vet., Injektionslösung	Sulfamethoxazolum_T MP	QJ01EW11	Injection	25	4	100
Trimethazol ad us. vet., Injektionslösung	Trimethoprim_sulfa	QJ01EW11	Injection	5	4	20
Tylan 200 ad us. vet., Injektionslösung	Tylosin	QJ01FA90	Injection	10	3	30
Tylan soluble ad us. vet., Arzneimittelvormischung	Tylosin	QJ01FA90	Premix	9	7	63
Tylucyl 200 ad us. vet., Injektionslösung	Tylosin	QJ01FA90	Injection	10	3	30
UFA 902 DUO ad us. vet., Arzneimittelvormischung	Sulfadimidin_TMP	QJ01EW30	Premix	20	10	200
UFA 902 DUO ad us. vet., Arzneimittelvormischung	Sulfathiazol_TMP	QJ01EW30	Premix	20	10	200
UFA 902 DUO ad us. vet., Arzneimittelvormischung	Trimethoprim_sulfa	QJ01EW30	Premix	8	10	80
ufamed Colistin 500 ad us. vet., Arzneimittelvormischung	Colistin	QA07AA10	Premix	6	10	60
Vesuprim ad us. vet., wässrige Injektionslösung	Sulfadiazin_TMP	QJ01EW10	Injection	20	4	80
Vesuprim ad us. vet., wässrige Injektionslösung	Trimethoprim_sulfa	QJ01EW10	Injection	4	4	16
Vetagent ad us. vet., Injektionslösung	Gentamicin	QJ01GB03	Injection	4	4	16
VITAL COLISTIN 125 ad us. vet., Arzneimittelvormischung	Colistin	QA07AA10	Premix	6	10	60
VITAL COLISTIN 2500 ad us. vet., Arzneimittelvormischung	Colistin	QA07AA10	Premix	6	10	60
VITAL COLISTIN 500 ad us. vet., Arzneimittelvormischung	Colistin	QA07AA10	Premix	6	10	60
Vital CST-222 ad us. vet., Arzneimittelvormischung	Chlortetracyclin	QJ01RA02	Premix	21	10	210
Vital CST-222 ad us. vet., Arzneimittelvormischung	Sulfadimidin	QJ01RA02	Premix	42	10	420
Vital CST-222 ad us. vet., Arzneimittelvormischung	Tylosin	QJ01RA02	Premix	3.6	10	36
Vital CST-222 L ad us. vet., Arzneimittelvormischung	Chlortetracyclin	QJ01RA02	Premix	21	10	210

Vital CST-222 L ad us. vet., Arzneimittelvormischung	Sulfadimidin	QJ01RA02	Premix	39	10	390
Vital CST-222 L ad us. vet., Arzneimittelvormischung	Tylosin	QJ01RA02	Premix	3.6	10	36
Vital TSS 480 ad us. vet., Arzneimittelvormischung	Sulfadimidin_TMP	QJ01EW30	Premix	20	10	200
Vital TSS 480 ad us. vet., Arzneimittelvormischung	Sulfathiazol_TMP	QJ01EW30	Premix	20	10	200
Vital TSS 480 ad us. vet., Arzneimittelvormischung	Trimethoprim_sulfa	QJ01EW30	Premix	8	10	80
Vital TSS 96 ad us. vet., Arzneimittelvormischung	Sulfadimidin_TMP	QJ01EW30	Premix	20	10	200
Vital TSS 96 ad us. vet., Arzneimittelvormischung	Sulfathiazol_TMP	QJ01EW30	Premix	20	10	200
Vital TSS 96 ad us. vet., Arzneimittelvormischung	Trimethoprim_sulfa	QJ01EW30	Premix	8	10	80
Westocillin ad us. vet., Injektionssuspension	Benzylpenicillinum procainum	QJ01RA01	Injection	10	2	20
Westocillin ad us. vet., Injektionssuspension	Dihydrostreptomycin	QJ01RA01	Injection	12.5	2	25
Zuprevo® 40 mg/ml ad us. vet.[A], Injektionslösung	Tildiprosin_LA	QJ01FA96	Injection	4	1	4

References:

DANMAP: DANMAP 2015: Use of antimicrobial agents and occurrence of antimicrobial resistance in bacteria from food animals, food and humans in Denmark F. Bager, K. Pedersen, RL. Skov, U. Wolff Sönsken: Statens Serum Institut,
National Veterinary Institute, Technical University of Denmark National Food Institute, Technical University of Denmark. 2016, http://www.danmap.org. Abrufdatum: 31.07.2017.

EMA: Principles on assignment of defined daily dose for animals (DDDvet) and defined course dose for animals (DCDvet); European Medicines Agency: EMA/710019/2014
Veterinary Medicines Division. 2015, http://www.ema.europa.eu. Abrufdatum: 31.0.2017.

EMA: Defined daily doses for animals (DDDvet) and defined course doses for animals (DCDvet); European Medicines Agency: EMA/224954/2016, Veterinary Medicines Division. 2016, http://www.ema.europa.eu. Abrufdatum: 31.07.2017.

MARAN: MARAN 2017: Monitoring of Antimicrobial Resistance and Antibiotic Usage in Animals
in the Netherlands in 2016; K.T. Veldman, D.J. Mevius, , B. Wit, , W. van Pelt, , D. Heederik: Wageningen Bioveterinary Research, Lelystad, Netherlands. 2017, http://www.wur.nl. Abrufdatum: 31.07.2017.

NORM-VET: NORM/NORM-VET 2015. Usage of Antimicrobial Agents and Occurrence of Antimicrobial
Resistance in Norway; G.S. Simonsen, A.M. Urdahl: Tromsø / Oslo,
Norway. 2016, http://www.vetinst.no. Abrufdatum: 31.07.2017.

TAK: Tierarzneimittelkompendium der Schweiz: Hrsg.: D. Demuth & C. Müntener; Institut für Veterinärpharmakologie und -toxikologie, Zürich, Schweiz. 2017, http://www.tierarzneimittel.ch. Abrufdatum 31.07.2017.

WHO: DDD: Definition and general considerations; Collaborating Centre for Drug Statistics Methodology: Norwegian Institute of Public Health, Oslo, Norway. 2016, http://www.whocc.no. Abrufdatum: 31.07.2017.

WHO: ATCvet Index 2016; Collaborating Centre for Drug Statistics Methodology: Norwegian Institute of Public Health, Oslo, Norway. 2017, http://www.whocc.no. Abrufdatum: 31.07.2017.
